# Asymbiotic seed germination and *in vitro* seedling development of *Orchis militaris*, an endangered orchid in Siberia

**DOI:** 10.1186/s43141-021-00223-1

**Published:** 2021-08-19

**Authors:** Aleksandra Yurievna Nabieva

**Affiliations:** Department of Biotechnology, Central Siberian Botanical Garden SB RAS, Zolotodolinskya str. 101, Novosibirsk, 630090 Russia

**Keywords:** Orchidaceae, Endangered species, Asymbiotic seed germination, Life cycle, Organic additives

## Abstract

**Background:**

Terrestrial orchids belonging to the *Orchis* genus are difficult to propagate and are under great pressure in their natural habitats. Studies regarding the influence of photoperiod and temperature regimes on *Orchis militaris* germination and morphological changes during immature seed development in vitro are scarce. Our aim was to identify photoperiod, temperature, and different nutrient media requirements for optimization of *O*. *militaris* seed germination and vigorous seedling production.

**Results:**

Post-germination morphological changes were recorded with *O*. *militaris* seeds collected from 32-day-old fruits, where the percentage of *O*. *militaris* seeds without embryo was 38.4%. The highest rate of *O*. *militaris* seed germination (82.6%) was obtained on Malmgren modified terrestrial orchid medium (mM), enriched by 5% coconut water, 5% birch sap, and 0.1% AC. Nine percent of seedlings were able to reach the advanced seedling stage (stage 6) after 12 months of maintenance on this medium. In all 3 modified media (Harvais, Knudson С and Malmgren), regeneration was via the production of protocorms and seedlings without callus formation. It was proved that more abundantly vigorous protocorms were formed on the modified Harvais 2 under continuous darkness, while the subculture in Knudson C medium with AC addition could be necessary to stimulate their further development. The regeneration success of the species for in vitro conditions was increased by following its natural seasonal cycle.

**Conclusion:**

This study demonstrated an efficient micropropagation system for *O*. *militaris* using immature seeds and thus widely opened the perspectives for its conservation in nature. The favorable conditions of seed germination periods for in vitro culture, identified as the definite shift of temperatures and photoperiod regimes intrinsic to the species in nature, could improve seedling survival of this medicinally important orchid.

## Background

The genus *Orchis* Tourn. ex L. (Orchidaceae) includes 21 accepted species and 16 subspecies [1, 2]. The species belonging to the genus are of great economic importance as their tubers are used to produce a hot beverage called salep, mainly consumed in Greece and Turkey [3]. It contains a nutritious starch-like polysaccharide—glucomannan, which is effective in curing sore throat, digestive problems, diarrhea, and gum disease [4]. The increasing popularity of traditional, organic food has led to a revival of salep consumption and overharvesting of the plants from the genus *Orchis* [5]. The species of the genus can be cultivated [6]; nevertheless, many orchids are collected rather from nature [3]. For the protection of rare and endangered orchid species, both in situ and ex situ approaches are important. Tissue culture collections became the most important tools in ex situ conservation of terrestrial orchids [7, 8]. Regardless of the studies conducted toward mass-propagation techniques involving some *Orchis* species [9, 10], tissue culture system for *O*. *militaris* propagation in vitro was undeveloped.

Furthermore, data on physiological and morphological aspects of *O*. *militaris* seed germination and development are scarcely provided, but they are urgently needed to establish in vitro collection and cryopreservation of the species.

We focused on the in vitro germination of the soldier’s orchid—*Orchis militaris* L., a cold-hardy terrestrial Eurosiberian species which has continental distribution, occurring from the Atlantic Coast to as far east as Mongolia [11]. As compared to other mainly subtropical representatives of the genus *Orchis*, *O*. *militaris* demonstrates a loss in total habitat and might be particularly negatively affected by drought because the species occupies the territories with the highest precipitation [12].

This endangered species is included in Annex II of the CITES International Convention and listed in the Red Data Book of the Russian Federation as a rare species found sporadically and in a small population [13]. Small-sized populations, as well as the clusters and groups of individuals that *O*. *militaris* usually form, might be due to the presence and diffusion of specific fungal symbionts, as suggested by [14].

*O*. *militaris* is a summer-winter tuberous perennial polycarpic orchid with spherical tuberoids on short stolons. Usually, two tuberoids can be found on the plant: one developed in the previous year (old tuber), and the second (younger one), developed from the basal part of the stem after flowering, consists of storage materials and a new bud for the next year. Plants consist of a rosette of leaves with a single inflorescence varying from 20 to 45 cm, with a single inflorescence bearing 10–40 hermaphroditic flowers [11]. The species is confined to calcareous soils and occurs more frequently in grazed damp and dry meadows, often among bushes or in light forests and along meadow slopes of mountains [1]. *O*. *militaris* does not tolerate mowing, cattle trampling, or recreation and under adverse conditions may pass for 3–8 years into a state of temporary underground rest [15]. In populations *O*. *militaris* multiplies mainly by seeds, but fruit set tends to be generally lower than 30% [16, 17]. It is proved that after a seed germinates it takes about 4 years before the orchid leaves appear, and four years more before the plant produces flowers [11, 18].

In order to assess the possibility of **in vitro** propagation of *O***.** *militaris*, effects of basal media plant growth regulators and natural additives on **in vitro** seed germination, protocorm development, and plantlet formation were studied. The aim of the study was to clarify the development stages of *O***.** *militaris* plants from germination until the formation of new regenerant under the different light and photoperiod regimes. **In vitro** propagation of *O***.** *militaris* from seeds should be considered as part of an integrated conservation program aimed at safeguarding the species from extirpation in the wild.

## Methods

### Orchid seed source

*O*. *militaris* immature seeds were collected from naturally pollinated plants approximately 32 days after anthesis in 2019 in the Novosibirsk Region (NR), Russia. When pollination occurred, the flower became wilting, and the days after pollination was accounted. *O*. *militaris* immature fruits were immediately used or stored in a light thermostat (Rumed, Germany) under a 16-h photoperiod with a light intensity of 20 μmol m^−2^ s^−1^ and a temperature of 7 °C for 1 day before the inoculation in vitro*.* Selecting the green capsules is widely used methodology for the successful germination in vitro for many terrestrial orchids, which mature seeds exhibited dormancy-related germination issues [19]. It was shown that their low percentage germination is attributed to high ABA levels and formation of a thick carapace with phenolic deposits during seed maturation [20]. The cenopopulation is located at 190 m elevation; N 54027′42′′; E 83020′42′′. Identification of species was done according to [21]. The territory, where *O*. *militaris* cenopopulation was found, belongs to the forest-steppe region of West Siberia. It occurred in post-forest successional meadow with scattered birch trees at the edge of the birch forest. Co-growing plant species at this location were represented by *Filipendula vulgaris* Moench. *Aegopodium podagraria* L., *Euphorbia virgata* Waldst. Et Kit., *Plantago maxima* Juss. ex Jacq., *Sanguisorba officinalis* L., *Phlomis tuberosa* L., *Pedicularis resupinata* L., *Galium boreale* L., *G. verum* L., *Medicago falcata* L., *Vicia cracca* L., *Cypripedium macranthon* SW., *Epipactis palustris* (L.) Crantz, *E. helleborine* (L.) Crantz, *Anemone sylvestris* L., *Ranunculus acris* L., and *Thalictrum simplex* L.

### Asymbiotic seed germination

The present research was conducted to study the *O*. *militaris* asymbiotic seed germination and seedling development under the influence of photoperiod, medium composition, and hormone treatments. Green undehisced capsules of *O*. *militaris* were collected from the medium part of each specimen’s inflorescence, washed under running tap water for 30 min, and submerged in a mixture of 2 drops of Tween-20 and 20% Domestos, a commercial detergent, for 10 min. Three replicates, each consisting of about 100 seeds, were counted. Thereafter, under aseptic conditions, capsules were treated with 0.25% mercuric chloride (w/v) for 8 min, then with 70% ethyl alcohol for 30 s, and flamed. Finally, fruits were rinsed 3 times with sterilized distilled water. The seeds were scooped out from the capsules and small masses of the aggregated seeds. The seeds from each capsule were equally distributed in 75 × 120 mm Petri dishes (~ 200 seeds/plate) containing 20 ml of 3 variants of sowing medium. Three replicate dishes were prepared for each accession of seeds. These initial media were based on a modified formula of Harvais [22] with or without addition of growth regulators (PGRs): (1) modified Harvais without PGRs—mHar0; (2) modified Harvais with 4.92 μM 2iP + 1.61 μM IBA—mHar2; (3) modified Harvais with 4.44 μM BA + 1.61 μM IBA—mHar3. The original medium was modified as follows: agar was reduced from 10 to 6 g l^−1^, and 10 g l^−1^ of sucrose was added. The addition of 100 g l^−1^ potato homogenate (PH) was implemented according to original receipt of Harvais medium. Percentages of germinated seeds inoculated onto 3 media variants: mHar0, mHar2, and mHar3 and cultured under 0/24 h L/D were counted in comparison with those cultured on the same media under 16/8 h L/D photoperiod (mHar0^*^, mHar2^*^, and mHar3^*^). For *O*. *militaris*, seed viability determination (the ratio of empty seeds to seeds with embryos) was calculated under stereo microscope in 10 different sights of view of each seed lot taken in experiment.

**Fig. 1:**
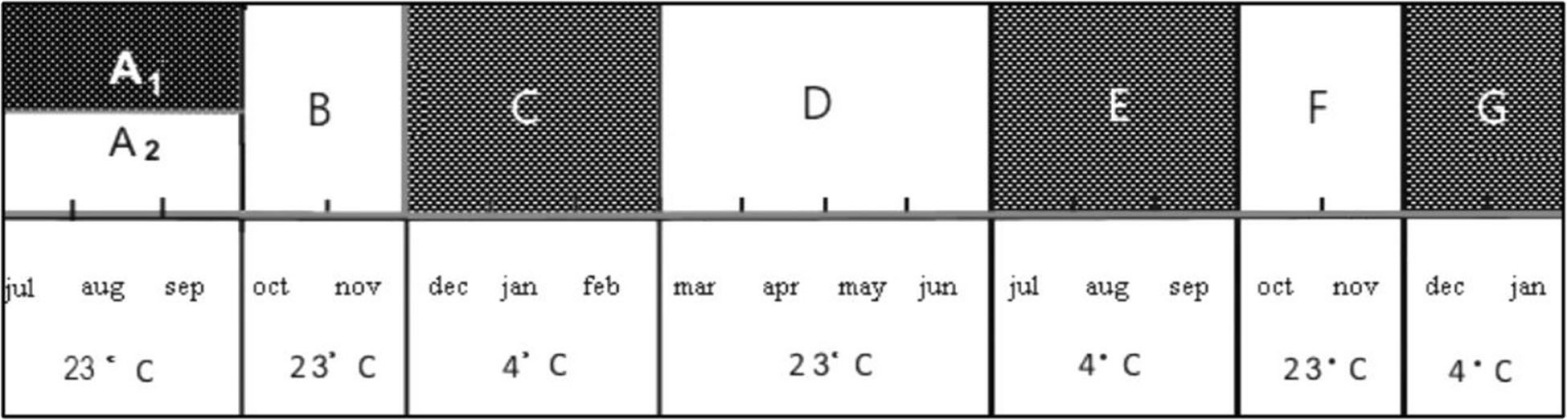
Scheme of experimental timing (July 2019–January 2021) followed in the study of *O*. *militaris* seedlings arising from immature seeds. # From the top to the foot of the dark and white rectangles (denoting dark and light conditions, applied during the periods of the orchid seed germination, marked as A_1_, A_2,_ B, C, D, E, F, G) are indicated: timing of the seed cultures (months); temperature conditions (°C)

Germination was recorded based on the number of full seeds, discounting seeds without embryos. Early seedling development was recorded up to a maximum of 16 months. Germinated seeds were counted monthly after sowing until germination by using stereo microscope (Stereo Discovery V 12, Carl Zeiss, Germany). The percentage of protocorms and seedlings in a developmental stage was calculated by dividing the number of seeds at that stage by the total number of germinated seeds. At the beginning of each cultivation period (Fig. 1), cultures were observed to determine the time of germination at 1-month and to reveal the appearance of protocorm developmental stages at 2-month intervals.

Seeds and developing protocorms were identified and assigned to one of developmental stages accordingly to study the development of seeds of other terrestrial orchids [23], where the stage 6 was added, which was distinguished by having two expanded leaves (Table 1). Seeds at stages 1 and 2 are considered not germinated. Germination (stage 3) indicated the emergence of the full embryo from the testa (Table 1).

**Table 1:** Description of developmental stages during orchid seeds germination, according [23] with some alterations concerning stage 2 (marked*)

Stage	Description
Stage 1	Unimbibed seed with intact testa
Stage 2*	Swelling of embryo and its enlargement followed by splitting of testa
Stage 3^1^	Protocorm arising, when rupture of testa occurred and first rhizoids developed
Stage 4^1^	Enlargement of protocorm and formation of protomeristem
Stage 5^1,2^	Further enlargement and development of first green leaf
Stage 6^1,2^	Emission of second green leaf

# ^1^ Stages considered as germinated; ^2^ photosynthetic stages

After germination at different photoperiods (Fig. 1(A_1_, A_2_)), all protocorms reaching 2 mm in size were transferred in 150-ml culture glass jars on Knudson С medium [24] without PGRs, modified by supplementing with 0.1% AC (mKC), for 2 months under cool white fluorescent light (Philips, Pila, Poland) at an intensity of 32 μmol s^−1^ m^−2^ with 16/8 h L/D photoperiod (Fig. 1B). For subsequent development, protocorms and seedlings were maintained for 13 months on 6 different hormone-free growing media: (1) mKС; (2) mKС with organic additives (OA) = mKC + OA; (3) double modified Harvais medium—DmHar; (4) DmHar with OA = DmHar + OA; (5) Malmgren [25] modified medium (mM); (6) mM with OA = mM + OA. The KC and M media modification consisted in the addition of the same contributions of micronutrients, vitamins, sucrose, and agar identical to the modified Harvais medium (mHar), as outlined above. A double modification of mHar medium was in the replacement of 100 g l^−1^ PH by the combination of OA: 5% coconut water (CW) and 5% birch sap (BS). All initial and growing media were adjusted to pH 5.8 before autoclaving.

The protocorms with meristematic center were maintained in the dark conditions at 4 °C for 3 months (Fig. 1C). During this period, the effects of cytokinin (BA or 2iP) applied with IBA and one of 2 illumination regimes on germination and protocorm growth were evaluated.

After leaf primordium generation, we applied 16-photoperiod during 4 months in combination with 23 ± 2 °C until the first leaf was formed (Fig. 1D) or the second one occurred (Fig. 1F). In the course of 16 months of the experiment, *O*. *militaris* seedlings were subjected to 3 dormant periods (Fig. 1C, E, G) (maintenance at 4 °C in dark conditions), alternated with 3 growing periods under 16 h/day light at 23 ± 2 °C (Fig. 1B, D, F). This *O*. *militaris* seed–protocorm–seedling growth cycle supported in vitro was similar to the natural life cycle of the species [11, 18].

Mean germination values (percentage of germinated seeds) were calculated by dividing the number of seeds at stage 3 by the total number of seeds with embryos. After 3 months of culture, the protocorm formation frequency was also calculated as the percentage of the number of protocorms with promeristem among the total germinated seeds; this value was also showing the amount of germinated seeds giving plantlets.

Each treatment had 3 replicates and was conducted three times. One-way analysis of variance (ANOVA) in STATISTICA 8 software (StatSoft Inc., Tulsa, OK) was used for statistical analysis. Seed germination and viability data were given as mean ± standard error (SE). Means were compared using Duncan’s Multiple Range Test (DMRT) at a significance level of *p* < 0.05.

## Results

In the study, propagation of *O*. *militaris* from seeds has been achieved through the use of fruits collected at 32 days after anthesis, which were green and un-dehisced. Immature seeds of *O*. *militaris* had light brown, nearly colorless testa (Fig. 5A) in contrast to mature seeds (60 days after anthesis), which have a dark brown, translucent one (Fig. 5B). There was no significant difference in the percentage of aborted *O*. *militaris* seeds counted for immature and for mature ones: 38.4 ± 2.5 and 40.0 ± 3.7, respectively.

**Fig. 2:**
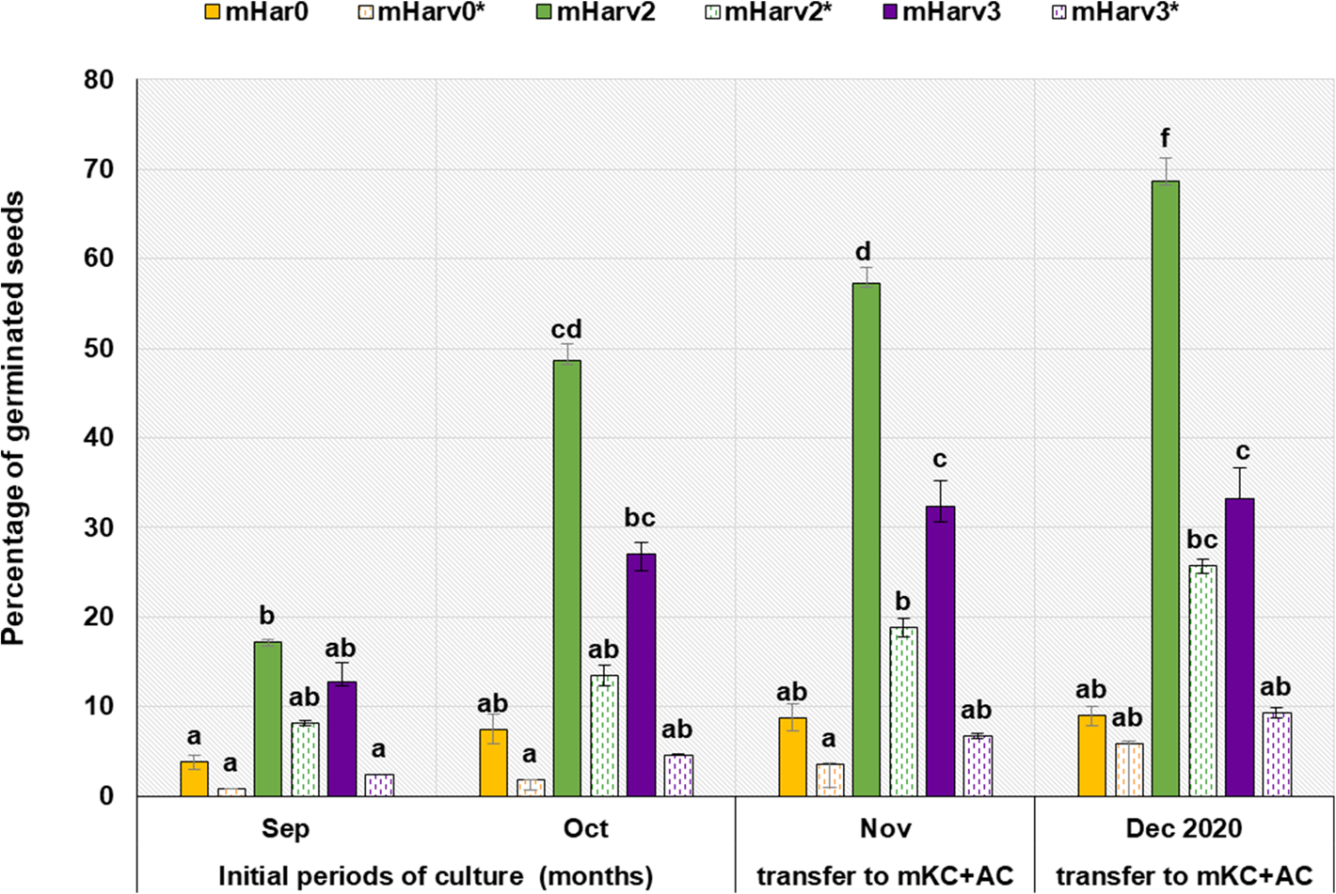
Comparative effects of plant growth regulators and photoperiod on the germination of immature seeds of *Orchis militaris* during 5 months of cultivation. **#** * mark is the indication of the 16/8 h L/D photoperiod conditions, in which seed cultivation on these media was performed, in contrast to the dark conditions (0/24 h L/D), applied with the media without this mark; the means having the same superscript letters in a column were not significantly different as determined by Duncan’s multiple range test (*p* < 0.05)

It was observed that *O*. *militaris* immature seeds were capable of germinating on all media tested regardless of illumination regime, but the rate of protocorms and seedlings development depended largely on the presence of definite cytokinin type and applied photoperiod (Fig. 2). All PGRs treatments under 2 photoperiod regimes, assayed for inducing asymbiotic *O*. *militaris* seed germination, generated the highest amount of protocormes. Only minimal germination was observed with the seeds cultivated on mHar0 medium without PGRs (less than 10%), whereas BA and 2iP application led to a significant increase in protocorm formation (27.0 % and 48.6%, respectively). The continuous darkness (Fig. 1(A_1_)) and 2iP mediated medium (mHar2) both resulted in a significantly higher number of protocorms (Fig. 2).

Subsequent transplantation was performed according to embryo growth rate and the stage of protocorm development. The most numerous protocorms, which reached stage 4 during maintenance on mKC with AC addition (68.7 ± 2.6%), were those originated from immature seeds, inoculated on mHar2 medium (Fig. 2). Most appropriate for transplanting were protocorms which had produced a leaf primordium (stage 4) and reached 2–3 mm Ø. For subsequent cultivation, only these protocorms transferred from mHar2 medium were used. When cultivated at 23 ± 2 °C on the media except mKC, supplemented with 0.1% AC, protocorms stopped growing in size, and browning of the medium was observed. Thus, intermediate period for 2 months of protocorm maintenance on mKC medium without PGRs but with 0.1% AC added was necessary to produce viable protocorms.

**Fig. 3:**
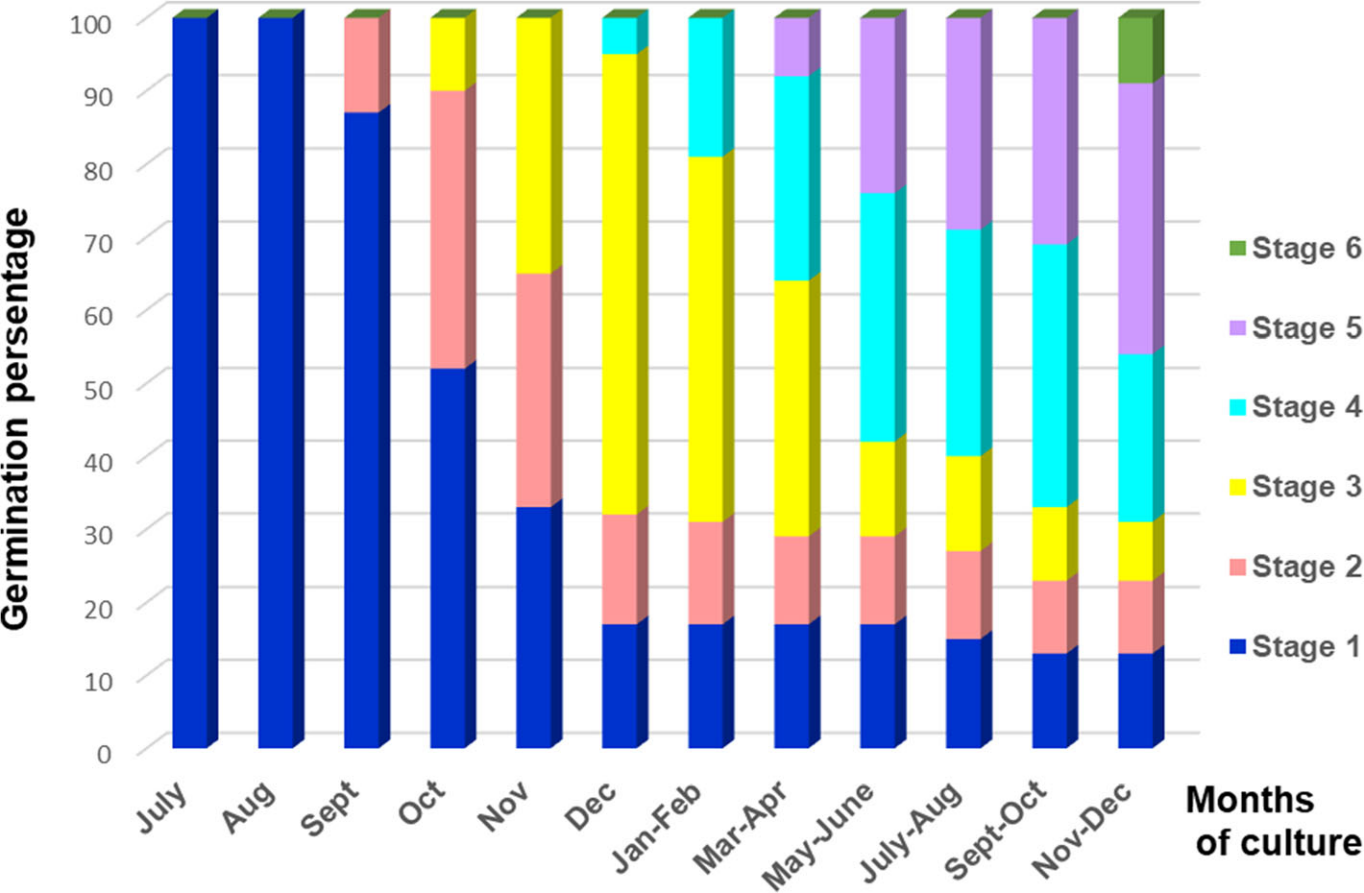
Germination percentage during different stages of *Orchis militaris* seed development scored monthly on mHar2 and mKC + AC media and 2-monthly on mM + OA medium

**Fig. 4:**
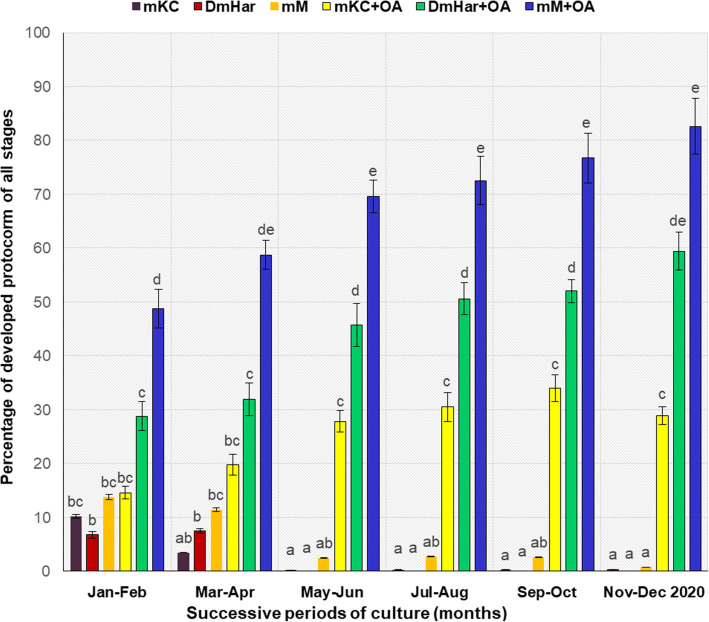
Effects of growing medium composition and organic additives on *Orchis militaris* protocorm formation. **#** Means having the same superscript letters in a column were not significantly different by Duncan’s multiple range test (*p* < 0.05)

In order to examine the effect of culture media for the successful protocorm and seedling development in the second part of the experiment, the efficiency of 6 growth media with or without organic additives (CW and BS) were tested. All media affected protocorm development differently: the highest protocorm development percentage was obtained on mM + OA medium (82.6%), followed by DmHar + OA (59.4%) and by mKC + OA (38.3%) (Fig. 4). The addition of organic compounds (CW and BS) to mM medium continued to have a higher number of protocorms and seedlings, which produced first secondary roots and true leaves (stage 6, Fig. 3; Fig. 4). Only protocorms firstly appeared on mHarv2 under continuous darkness and further maintained on mKC + AC and on mM + OA media were able to reach the advanced seedling stage (stage 6) within 16 months of culture (Fig. 3).

MM medium, supplemented with the organic compounds (CW and BS) showed the highest efficiency for protocorm formation (in comparison with other media with the same additives (Fig. 4). When protocorms (2 mm and more in Ø) were transferred for cultivation on the growing media, which were not supplemented with OA, advanced seedling maintenance was not achieved, protocorm and plantlet development finally failed. The data obtained indicate the beneficial effect of organic additives including CW and birch phloem sap, which enhanced the appearance of *O*. *militaris* seedlings from protocorms.

**Fig. 5:**
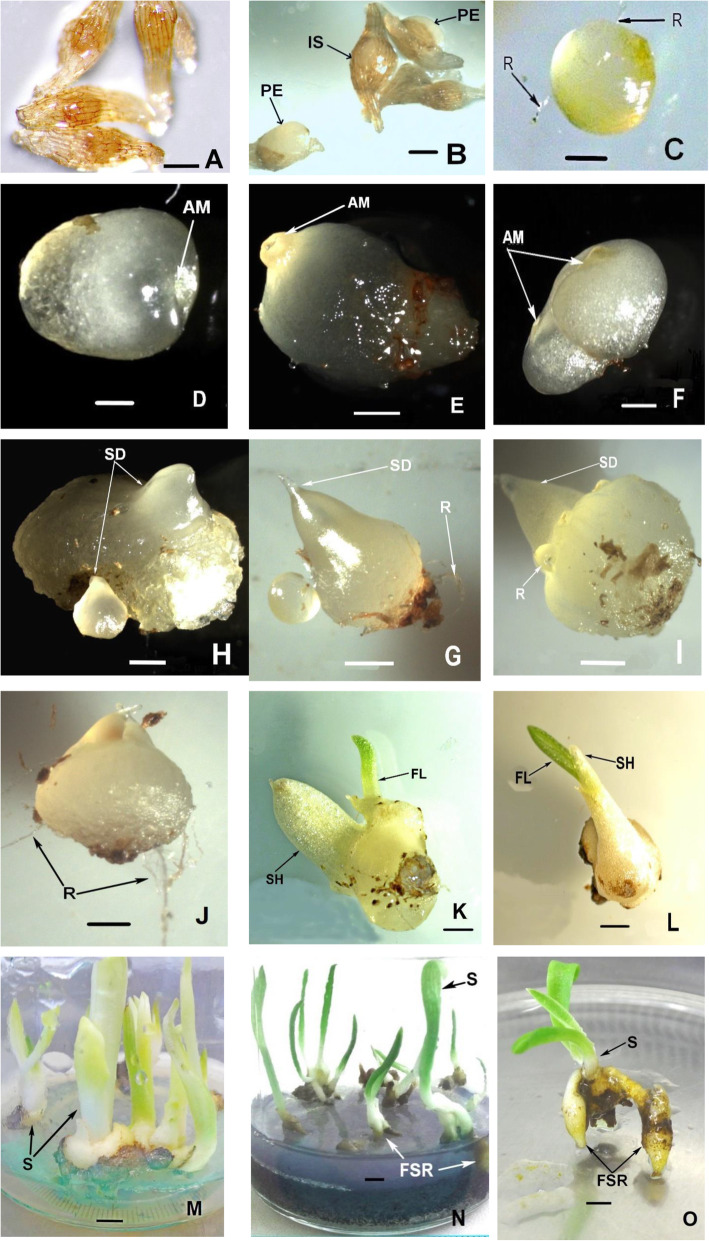
The successive developmental stages of *Orchis militaris* seed germination in asymbiotic culture in vitro for 16 months. **A** Stage 1: immature seeds with intact testa. **B** Stage 2: imbibed seed (IS) and seeds with swollen embryo in the width of seed coat, splitting of testa followed by protocorm emergence (PE)—Stage 3. **C** Stage 3: rhizoids (R) are formed on protocorm. **D** Stage 4: protocorm enlargement with apical meristem (AM) sinking. **E** Stage 4: superficial disc grows into a conical meristematic dome. **F** Stage 4: protocorm possesses 2 centers of meristematic activity. **H** Population of protocorms at various stages of shoot apex differentiation (SD). **G** Stage 5: protocorm with extended shoot differentiation (SD). **I**, **J** Stage 5: rhizoids formed on the basal part of the protocorm became elongated. **K** Stage 5: seedling (**S**) with the first leaf (FL) emergence and the long sheath (SH) primordium initiating in its anterior part. **L** Stage 5: at the early seedling stage the elongation of the first leaf was observed. **M**, **N** Stage 6: seedlings (**S**) obtained in vitro before transplanting in soil conditions. **O** Stage 6: advanced seedling stage distinguished by two and more expanded leaves and the developed first secondary roots (FSR). # **A**, **B**—scale bar 100 μm; **C**, **D**, **E**, **F**, **H**—scale bar 200 μm; **G**, **I**, **J**, **K**—scale bar 500 μm; **L**, **M**, **N**, **O**—1 mm

The seeds sown on July 14 started to germinate in mid-October. The first stage of germination was the imbibition of water by the seed, which led to omnidirectional embryo enlargement. Initially the embryos became white, and globular embryo was formed (Fig. 5B). The globular stage subsequently developed into the swelling stage; the first protocorms appeared after 2 months of *O*. *militaris* seeds inoculation (Fig. 5C). The early phase of protocorm formation commenced with the appearance of the apical meristem dome and the few translucent rhizoids (Fig. 5C, E). The protocorm formation depends on the type of culture medium used. In our study, the highest rate of protocorm and seedling formation in *O*. *militaris* was obtained on Malmgren modified terrestrial orchid medium (82.4%).

Longitudinal growth at the chalazal pole followed, as in other terrestrial orchid species [26], and the apical dome subsided into the body of the protocorm (Fig. 5D). Protocorms of *O*. *militaris* exhibited a phase of conical meristematic dome growth without the production of photosynthetic leaf primordia (Fig. 5G, I). This phase (Fig. 5J) is also characterized by prolific rhizoid production. The stage of seedling began when the first leaf appeared and a stem developed (Fig. 5K), and plantlets were then suitable to move into light; after that, the apical dome turned green within 2–3 weeks (Fig. 5L). The development of protocorms into plantlets was normal (without callus formation).

True leaves and roots were observed 14–15 months after germination, but root initiation on all media was yet limited, and a maximum of 24.5% was recorded for *O*. *militaris* seedlings cultured on mM + OA media. The advanced *O*. *militaris* seedling stage (Fig. 5M, N, O) was distinguished by multiple leaves and roots development. Nine percent of plantlets, which have 4.0–6.5 cm long shoots and roots, formed when the second leaves appeared after the additional 2-month cold treatment (4 °C), were appropriate for transplanting into soil after 16 months from the inoculation (Fig. 5O).

The most suitable way to obtain *O*. *militaris* seedlings reaching stage 6 of development appeared to be seed incubation for 3 months on mHar2 medium with 4.92 μM 2iP + 1.61 μM IBA, followed by 2-month maintenance on mKC with AC addition and subsequent cultivation on mM + OA medium without PGRs throughout shifting temperature and photoperiod regimes, according to the lifespan of the species (Figs. 1 and 3). Moreover, it was noted that despite showing early signs of germination, light-incubated seeds were unable to develop into seedlings.

## Discussion

*Orchis* species have been found to be more troublesome [27], in contrast with the easily in vitro propagated genera like *Dactylorhiza*. Researchers have observed low levels of *O*. *militaris* mature seed germination, especially during the first 7–8 months [28, 29]. In our study, the percentage of aborted *O*. *militaris* seeds (38.4%) was quite similar to the measurement carried out by [30] for naturally pollinated specimen (45.6%). The relatively low level of potentially competent (viable) seeds in comparison with rewarding species appears to be an intrinsic feature of the deceptive orchids like *O*. *militaris*, since they have no pollinator abundance [31].

It was demonstrated previously that selecting “green” capsules at a certain time of their development increases the rate of asymbiotic seed germination [32]. Thus, immature orchid seeds which have underdeveloped and permeable testa were successfully used as source material for our work.

Our previous results showed that the cultivation of *O*. *militaris* protocorms under the constant physical conditions on the same medium was unsuccessful: most protocorms stopped their growth at the early stage of swelled embryos (data not shown). These findings are in concordance with the data obtained by [33], showing that the regeneration success of orchids for in vitro conditions could be increased by following their natural seasonal cycle, where temperature and light changes are the significant requirements. Baskin et al. [34] recommended alternating temperatures during germination, as constant temperatures are not common in nature. During the life cycle in nature, early flowering *Orchis* species require 2–6 months’ dormancy period to generate basal leaves [11, 18, 35]. In this study, we attentively followed the *O*. *militaris* dormancy periods inherent in the orchid with a lifespan of 16 months, and thus observed the emergence of new developmental stages of seed germination just after the dormancy period was over. Similar data were reported for Mediterranean species *Orchis patens*, whose protocorms and seedlings were not able to grow successfully under invariably warm or cold conditions [36]**.**

In our study, germination of *O*. *militaris* immature seeds occurred regardless of photoperiod or media treatments; however, stage 6, which reflects advanced protocorm to seedling development, was exhibited only by the seeds initially incubated in the dark. Numerous studies showed that seed germination in many temperate terrestrial orchids is inhibited by light incubation [37, 38]. According to [39], the inhibition of germination of *Cephalanthera falcate* immature seeds was also endured by illumination. In this work, *O*. *militaris* seeds subjected to total darkness germinated more vigorously than those incubated in the light, as opposed to the findings of [9, 10], where the best photoperiod regime for germination of some *Orchis* species was indicated as 16/8 light/dark. This may suggest the lower light requirement for *O*. *militaris* immature seeds germination, in comparison with the *Orchis* species germinated by mature seeds.

This study provides evidence of the widely supported fact that the medium composition, where the mineral salt concentration has been reduced and organic compounds increased, had an important role in orchid seed germination and protocorm development [40]. Malmgren [25] reported on the benefits of reduced inorganic salts in the new basal medium, where organic additive such as PH was introduced. Similar to these data, the current study proved that the seeds of *O*. *militaris* germinate successfully in the presence of organic additives, in particular PH, CW, and BS. The selection of these OA was inspired by the results of [41], who showed that in the absence of CW, BS, or PH [42] the protocorm development was not observed in the course of *Cypripedium* seed germination. Based on the results of [43], who successfully applied PH in combination with CW for enhancement of *Cymbidium findlaysonianum* plantlet formation, we used only PH for initial germination of *O. militaris* seeds, while CW in combination with BS were applied for protocorm and seedling maintenance.

Organic supplements provide a natural source of carbohydrates, inorganic ions, amino acids, vitamins, and phytohormones, promoting growth and morphogenesis in orchid seed cultures. CW is known to support cell division in the tissue culture of orchids for its zeatin and zeatin ribosides content [44], whereas auxin and gibberellin-like constituents, found in BS [45], as well as carbohydrates and amino acids intrinsic to PH [46], commonly facilitate seed germination and enhanced seedling growth of tropical [47] and temperate [48] orchid species.

In many reports, PGRs proved to be effective in enhancing immature orchid seed germination: cytokinins promoted cell division of developing embryos [19], while auxins influenced leaf formation and protocorm morphogenesis [49]. Our observations showed that in the mHar0 treatment, excluding PGRs, nearly all protocorms stopped their growth at early developmental stages and this is in agreement with the results of [50], who obtained that protocorms were larger after two months of growth on the medium with 10 mg/l cytokinin, compared to other variants of germination study. The enhanced formation of *O*. *militaris* protocorms, growing to over 2 mm in height during 3 months, occurred at 23 ± 2 °C in darkness with the most effect of combination of 2iP and IBA treatment (in concentrations of 4.92 μM and 1.61 μM, respectively). The combination of these factors reflects the complexity of specific requirements for in vitro seed germination, which appeared to be unique for any orchid species. This advantage of the PGRs inclusion in basal medium is in agreement with the results of studies indicating that a combination of auxin and cytokinin was the most effective at promoting germination in orchid “green pod cultures” [51, 52]. In addition*,* [53] reported that a combination of IBA and BA improved protocorm recovery from endangered orchid of the genus *Orchis* and stimulated protocorm formation in *Serapias vomeracea* [54]. The early development of *Dactylorhiza* species seedlings was also enhanced by a combination of cytokinin 2iP with auxin [55]. Similar to these findings, our data confirmed the benefit of PGRs addition only at the initial stages of *O*. *militaris* seed germination, while the most favorable medium for protocorm enlargement and seedling development was mM + OA medium without PGRs, but supplemented with CW and BS.

We also detected the promotional effect of AC on *O*. *militaris* morphogenesis, which may be due to its adsorption of inhibitory phenolic compounds, often observed during the development of protocorms [56]. Thus, the subculture in the mKC with the addition of AC could be necessary to stimulate the development of protocorms. We suppose that AC was not necessary during the early (S1 and S2) stages of seed germination but could play an important role in *O*. *militaris* seedling development toward rhizogenesis, as has been reported for another orchid species [57]. Our results showed that, even in the absence of auxin, root development occurred in shoots grown on mM medium containing AC. Thus, mM + OA, which combined AC and complex OA, was found to be the most suitable medium for the formation of *O*. *militaris* plantlets from protocorms.

## Conclusion

Optimization of different temperature and light regimes for the in vitro germination and culture of *O*. *militaris*, comparable to seasonal temperature changes and dormant periods which the species endured under the natural conditions, was performed. Seeds cultured under 0/24-h light/dark photoperiod were capable of evolving through subsequent development stages, when protocorms could grow to over 2 cm in height following 2 months of culture on mHar2 sowing medium supplemented with 4.92 μM 2iP +1.61 μM IBA, followed by mKC, supplemented with 0.1% AC and then by Malmgren modified medium, enriched by 5% CW, 5% BS, and 0.1% AC. *O*. *militaris* protocorms maintained on mM ± OA medium successfully progressed toward the advanced seedling stage (stage 6) within 12 months. Hence, this efficient procedure for regenerating protocorms and seedlings from immature seeds of *O*. *militaris* could be used for commercial exploitation and *ex situ* conservation of this valuable endangered terrestrial orchid species.

## Data Availability

Not applicable.

## Abbreviations

BA: 6-Benzylaminopurine
IBA: Indole butyric acid
2iP: 2-Isopentenyladenine
mHar: Harvais medium
mKC: Knudson C medium
mM: Malmgren medium
PGRs: Plant growth regulators
OA: Organic additives
PH: Potato homogenate
AC: Activated charcoal
CW: Coconut water
BS: Birch sap

## References

[1] Kretzschmar H, Eccarius W, Dietrich H (2007). The orchid genera Anacamptis.

[2] Govaerts R, Bernet P, Kratochvil K, Gerlach G, Carr G, Alrich P, Pridgeon AM, Pfahl J, Campacci MA, Baptista HD, Tigges H, Shaw J, Cribb PJ, George A, Kreuz K, Wood J (2016) World Checklist of Orchidaceae. Royal Botanic Gardens, Kew, London. Available at http://apps.kew.org/wcsp/

[3] Kreziou A, De Boer H, Gravendeel B (2016). Harvesting of salep orchids in north-western Greece continues to threaten natural populations. Oryx.

[4] Jagdale SP, Shimpi S, Chachad D (2009). Pharmacological studies of 'Salep'. J. Herb Med Toxic.

[5] Ghorbani A, Gravendeel B, Naghibi F, de Boer HJ (2014) Wild orchid tuber collection in Iran: a wake-up call for conservation. Biodivers Conserv 23: 2749–2760.

[6] Seaton P, Ramsay M (2005). Growing orchids from seed.

[7] Jakobsone G (2008). Morphogenesis of wild orchid *Dactylorhiza fuchsii* in tissue culture. Acta Univer Latv, Biology.

[8] Valletta A, Attorre F, Bruno F, Pasqua G (2008). *In vitro* asymbiotic germination of *Orchis mascula* L. Plant Biosyst.

[9] Bektas E, Cuce M, Sokmen A (2013). *In vitro* germination, protocorm formation, and plantlet development of *Orchis coriophora* (Orchidaceae), a naturally growing orchid species in Turkey. Turk J Bot.

[10] Farrell L (1985). Biological flora of the British Isles: *Orchis militaris* L. J Ecol.

[11] Evans A, Janssens S, Jacquemyn H (2020) Impact of climate change on the distribution of four closely related Orchis (Orchidaceae) species. Diversity 12(8), 312. 10.3390/d12080312.

[12] Averyanov LV, Bardunov LV, Novikov VS (2008). *Orchis militaris* L. The Red Data Book of the Russian Federation (Plants and Fungi).

[13] Calevo J, Voyron S, Ercole E, Girlanda M (2020) Is the distribution of two rare *Orchis* sister species limited by their main mycobiont? Diversity 12(262). 10.3390/d12070262

[14] Fardeeva MB (2007) Ontogeny of *Orchis militaris* L. In: Ontogenetic Atlas of Plants. 5:238–241. Mari. Gos. Univ, Yoshkar Ola.

[15] Tremblay RL, Ackerman JD, Zimmerman JK, Calvo RN (2005) Variation in sexual reproduction in orchids and its evolutionary consequences: a spasmodic journey to diversification. Biol J Linn Soc 84:1–54. 10.1111/j.1095-8312.2004.00400.x

[16] Henneresse T, Wesselingh RA, Tyteca D (2017) Effects of floral display, conspecific density and rewarding species on fruit set in the deceptive orchid Orchis militaris (Orchidaceae). Plant Ecol. Evol 150:279–292. doi.org/10.5091/plecevo.2017.1313.

[17] Vakhrameeva MG, Varlygina TI, Tatarenko IV (2014). Orchids of Russia (biology, ecology and protection).

[18] Pierce S, Cerabolini BEL (2011). Asymbiotic germination of the White Mountain Orchid (Pseudorchis albida) from immature seed on media enriched with complex organics or phytohormones. Seed Sci Technol.

[19] Kendon JP, Rajaovelona L, Sandford H, Fang R, Bell J, Sarasan V (2017). Collecting near mature and immature orchid seeds for ex situ conservation: 'in vitro collecting' as a case study. Bot Stud..

[20] Krasnoborov IM (2000). Determinant of plants of the Novosibirsk region.

[21] Harvais G (1973). Growth requirements and development of *Cypripedium reginae* in axenic culture. Canad J Bot.

[22] Swarts ND, Dixon KW (2017). Conservation methods for terrestrial orchids.

[23] Knudson L (1946). A nutrient for germination of orchid seeds. Am Orchid Soc Bull.

[24] Malmgren S (1996) Orchid propagation: theory and practice. In: Allen С (ed) Proceedings of North American native terrestrial orchid conference, Maryland.

[25] Rasmussen HN (1995). Terrestrial orchids: From seed to mycotrophic plant.

[26] Malmgren S, Nyström H (2011) Orchid Propagation. http://www.lidaforsgarden.com/Orchids/engelsk.htm

[27] Kulikov PV, Phillippov EG (1998). On propagation of temperate zone orchids by tissue culture methods. Bulletin of the Main botanical garden of Russian Academy of Science.

[28] Metsare M, Ilves A, Haldna H, Kull T, Tali K (2015). Four seed-quality measures in orchids with different pollination systems. Acta Bot Gallica.

[29] Ye Z-M, Dai W-K, Jin X-F, Gituru RW, Wang Q-F, Yang C-F (2014) Competition and facilitation among plants for pollination: can pollinator abundance shift the plant-plant interactions? Plant Ecol 215:3–13. 10.1007/s11258-013-0274-y

[30] Yeung EC (2017). A perspective on orchid seed and protocorm development. Bot Stud..

[31] Hürkan YK, Hürkan K, Aki C (2018). Comparative growth media performances on *in vitro* propagation of some salep orchids. Anadolu University Journal of Science and Technology - C Life Sciences and Biotechnology.

[32] Baskin CC, Thompson K, Baskin JM (2006). Mistakes in germination ecology and how to avoid them. Seed Sci Res.

[33] Delforge P (2006). Orchids of Europe, North Africa and the Middle East.

[34] Calevo J, Bazzicalupo M (2020) Less is more: low-cost *in vitro* . Nat Conserv Res 5(1):172–177. 10.24189/ncr.2020.043

[35] Arditti J. (ed) (1981). Orchid Biology. Reviews and perspectives. Orchid seed germination and seedling culture, vol 11. Cornell University Press, Ithaca, New York

[36] Teixeira da Silva JA (2013). Orchids: Advances in tissue culture, genetics, phytochemistry and transgenic biotechnology. Floricult Ornam Biotech.

[37] Yamazaki J, Miyoshi K (2006). In vitro asymbiotic germination of immature seed and formation of protocorm by Cephalanthera falcata (Orchidaceae). Ann Bot.

[38] Yeung EC, Li YY, Lee YI, Lee YY, Yeung EC (2018). Understanding seed and protocorm development in orchids. Orchid Propagation: from laboratories to greenhouses – methods and protocols.

[39] Huh YS, Lee JK, Nam SY, Paek KY, Suh GU (2016). Improvement of asymbiotic seed germination and seedling development of *Cypripedium macranthos* Sw. with organic additives. J Plant Biotechnol.

[40] Burch RM, Chambers CB (2013). Micropropagation and establishment of *Cypripedium* 'Lady slipper' orchids. Acta Hortic 988:161-166 10.17660/ActaHortic.2013.988.18

[41] Tawaro S, Suraninpong P, Chanprame S (2008). Germination and regeneration of Cymbidium findlay sonianum Lindl. on a medium supplemented with some organic sources. Walailak J Sci Tech.

[42] Tan S, Yong J, Ge L (2014). Analyses of phytohormones in coconut (*Cocos nucifera* L.) water using capillary electrophoresis-tandem mass spectrometry. Chromatography.

[43] Kefeli V, Kalevitch MV (2003) Natural growth inhibitors and phytohormones in plants and environment. In: Borsari B (ed) Kluwer Academic Publishers, Dordrech, p.324.

[44] Molnar Z, Virag E, Ordog E (2011). Natural substances in tissue culture media of higher plants. Acta Biol Szegedvol.

[45] Pakum W, Watthana S, Srimuang KO, Kongbangkerd A (2016). Influence of medium component on in vitro propagation of Thai’s endangered orchid: *Bulbophyllum nipondii* Seidenf. Plant Tissue Cult Biotechnol.

[46] Zhang Y, Lee YI, Deng L, Zhao S (2013). Asymbiotic germination of immature seeds and seedling development of Cypripedium macranthos Sw., an endangered Lady’s slipper orchid. Sci Hortic.

[47] Novak SD, Luna LJ, Gamage RN (2014). Role of auxin in orchid development. Plant Signal Behav.

[48] Ponert J, Vosolsobě S, Kmecová K, Lipavská H (2011). European orchid cultivation – from seed to mature plant. Eur J Environ Sci.

[49] De KK, Majumdar S, Sharma R, Sharma B (2006). Green pod culture and rapid micropropagation of Dendrobium chrysanthum Wall. – a horticultural and medicinal orchid. Folia Hortic.

[50] Godo T, Komori M, Nakaoki E, Yukawa T, Miyoshi K (2010). Germination of mature seeds of *Calanthe tricarinata* Lindl., an endangered terrestrial orchid, by asymbiotic culture *in vitro*. In Vitro Cell Dev Biol - Plant.

[51] Baker A, Kaviani B, Nematzadeh G, Negahdar N (2014). Micropropagation of *Orchis catasetum* – a rare and endangered orchid. Acta Sci Pol, Hortorum Cultus.

[52] Bektas E, Sokmen A (2016) In vitro seed germination, plantlet growth, tuberization, and synthetic seed production of Serapias vomeracea (Burm.f.) Briq. Turk J Bot 40:584–594 10.3906/bot-1512-13

[53] Novotna KW, Vejsadova H, Kindlmann P (2007). Effects of sugars and growth regulators on *in vitro* growth of *Dactylorhiza* species. Biol Plantarum.

[54] Gutierrez-Sanchez A, Monriboc-Villanueva JL, Cocotle-Ronzón Y, Martinez-Cruz NS, Guerrero-Analco JA (2020) Phenolic profile and antioxidant activity from wild and *in vitro* cultivated *Rhynchostele rossii (*Orchidaceae). Acta Bot Mex 127(127), e1665. 10.21829/abm127.2020.1665

[55] Vudala SM, Ribas LLF (2017) Seed storage and asymbiotic germination of *Hadrolaelia grandis* (Orchidaceae). S Afr J Bot 108:1–7. 10.1016/j.sajb.2016.09.008

[56] Merritt DJ, Hay FR, Swarts ND, Sommerville KD, Dixon KW (2014). *Ex situ* conservation and cryopreservation of orchid germplasm. Int J Plant Sci.

[57] Vujanovic V, St-Arnaud M, Barabe D, Thibeault G (2000). Viability testing of orchid seed and the promotion of colouration and germination. Ann Bot.

